# Complete genome sequence of Aquificaceae bacterial strain NHSK-567, isolated from Nakabusa Hot Springs, Japan

**DOI:** 10.1099/acmi.0.001222.v4

**Published:** 2026-11-05

**Authors:** Keisuke Kubota, Shigeru Kawai, Katsumi Matsuura, Shawn Erin McGlynn

**Affiliations:** 1 Earth-Life Science Institute, Institute of Science Tokyo, Meguro, Tokyo, Japan; 2 Toyohashi University of Technology, Toyohashi, Aichi, Japan; 3 Institute for Early Metabolic Evolution, Ota, Tokyo, Japan; 4 Blue Marble Space Institute of Science, Seattle, WA, USA; 5 RIKEN Center for Sustainable Resource Science, Wako, Saitama, Japan

**Keywords:** *Aquificaceae*, *Hydrogenobacter*, terrestrial hot spring, thermophile, *Thermocrinis*

## Abstract

We report the complete genome sequence of *Aquificaceae* bacterial strain NHSK-567 isolated from Nakabusa hot springs. The genome consists of 1.47 Mb with a 46.1% guanine–cytosine (GC) content.

## Data Summary

The genome sequence of NHSK-567 has been deposited at DDBJ/ENA/GenBank under the accession number JCCFYS000000000. Raw sequence reads are available in the NCBI Sequence Read Archive under accession number SRR40236450.

## Introduction

The family *Aquificaceae* comprises thermophilic to hyperthermophilic bacteria found in terrestrial geothermal sites and marine hydrothermal systems worldwide [1, 2]. The family consists of six genera based on validly published names under the ICNP/LPSN, including *Aquifex*, *Thermocrinis*, *Hydrogenobaculum*, *Hydrogenobacter*, *Hydrogenivirga* and *Pampinifervens* [3, 4]. Members of this family are primarily capable of chemolithoautotrophic growth, utilizing inorganic compounds as electron donors and CO_2_ as the sole carbon source [4–6].

Nakabusa hot springs in Nagano, Japan, are slightly alkaline and sulphide-rich hydrothermal springs, where microbial mats form across a range of thermal and chemical gradients [7, 8]. As in other terrestrial geothermal sites, chemosynthetic bacteria, such as the *Aquificaceae*, have been reported to inhabit geothermal water streams and pools in this area at temperatures above 70 °C [9]. However, these bacteria have not yet been isolated, thereby hindering a comprehensive understanding of their ecology, physiology and interactions with the geothermal environment.

In this study, we successfully isolated *Aquificaceae* strain NHSK-567 from a microbial mat at Nakabusa Hot Springs (Kassen-no-yu) in Japan. To begin elucidation of the physiological traits and compare the metabolic differences from closely related species, the complete genome of the strain was sequenced.

## Methods

Black-coloured microbial mats were collected from a stream site at 85 °C (36° 23′ 33.3″ N 137° 44′ 53.0″ E) and stored in 50 ml plastic tubes filled with the hot spring water. A partial microbial mat sample was inoculated in a 50 ml glass vial, with modified low-salt medium containing ammonium chloride as the nitrogen source, K_2_HPO_4_ and KH_2_PO_4_ as the phosphate source and buffer, ferric ammonium citrate in place of FeCl_3_ and l-cysteine as a reducing agent [10]. Yeast extract and sodium pyruvate were supplemented in small amounts to enhance growth. H_2_, sodium thiosulphate and sodium sulphide were added as potential electron donors for chemolithotrophic growth. After a few days of cultivation at 85 °C under microaerobic conditions with H_2_, N_2_, O_2_ and CO_2_ in the gas phase, the medium colour changed from transparent to translucent, indicating cell growth. The strain was isolated by two rounds of dilution to extinction under the same conditions. The genomic DNA of the strain was extracted and purified using a Zymo soil/faecal DNA extraction kit (Zymo Research, Irvine, CA) and quantified with a NanoDrop 2000 (Thermo Fisher Scientific, Massachusetts, USA). The purity of the isolate was confirmed by 16S rRNA gene amplicon sequencing with the primer set of AGRGTTYGATYMTGGCTCAG and RGYTACCTTGTTACGACTT (27F/1492R), using PacBio Vega at Integrated Microbiome Resource (IMR), Dalhousie University (Nova Scotia, CA).

For genome sequencing, the DNA was submitted to IMR. The long-read library was prepared using PacBio SMRTbell Prep Kit 3.0. Genome sequencing was performed with a platform, PacBio Vega. The quality of the HiFi reads was assessed using FastQC (ver. 0.12.1) and NanoPlot (ver. 1.47.1) [11, 12]. The reads were assembled, circularized and polished with Flye (ver. 2.9.6) [13]. The circularization of the assembled genome was confirmed with Bandage (ver. 2022.09) [14]. Completeness and contamination of the assembly were evaluated with CheckM2 (ver. 1.1.0) [15]. To evaluate the base-level accuracy of the assembly, the consensus quality value (QV) was calculated using Merqury (ver. 1.3), based on the k-mer spectrum [16]. A k-mer database (k=15) was generated from the raw PacBio HiFi reads using Meryl (ver. 1.3) [16]. To assess the structural integrity and read support of the assembly, raw reads were aligned back to the assembled genome using minimap2 (ver. 2.31) [17]. The resulting alignments were sorted, and read coverage and mean mapping quality (MAPQ) were calculated using SAMtools (v1.23.1) [18]. All bioinformatic analyses were performed on the Galaxy platform (UseGalaxy.org), and default parameters were applied for all software unless otherwise specified [19]. The circular chromosome sequence was reoriented to begin upstream of the *dnaA* gene using the DFAST pipeline (ver. 1.6.0) [20]. Gene prediction and annotation were performed using the NCBI Prokaryotic Genome Annotation Pipeline (ver. 6.11) [21].

The 16S rRNA gene sequence similarity between strain NHSK-567 and its closest relatives was calculated using the Pairwise Nucleotide Sequence Alignment for Taxonomy tool in EzBioCloud [22]. Digital DNA–DNA hybridization (dDDH) values among the closely related type strains were calculated using Formula 2 of the Genome-to-Genome Distance Calculator (GGDC) on the Type (Strain) Genome Server (TYGS) [23]. Pairwise orthologous average nucleotide identity (orthoANI) values were calculated on the EzBioCloud web server [24].

## Announcement

The assembled genome of strain NHSK-567 was 1.47 Mb with a 46.1% GC content. Additional assembly and sequencing information are summarized in Table 1. The genome of strain NHSK-567 was assembled into a single circular chromosomal contig. The assembly graph is available in Supplementary Material 1 (available in the online Supplementary Material). An overview of the Galaxy workflow is provided in Supplementary Material 2.

**Table 1.** Assembly and genomic information for NHSK-567

Information	NHSK-567
Genome assembly accession	JCCFYS000000000
SRA (Sequence Read Archive) accession	SRR40236450
Genome size (bp)	1,471,742
GC content (%)	46.1
Number of assembled contigs	1
Raw read N50 (bp)	9,055.0
Number of raw reads	92,750
Total bases of raw reads (bp)	698,227,777
Completeness (%)^*^	99.99
Contamination (%)^*^	0.15
QV for assembly^†^	+inf
Breadth of coverage (%)^‡^	100
MAPQ^‡^	60
Genome coverage^§^	474.4
Number of coding sequences	1,586

* Completeness and contamination percentages were calculated using CheckM2 (ver. 1.1.0) based on its default machine learning prediction model.

† Quality value of the assembly was calculated with Merqury (ver. 1.3). The QV score +inf indicates that no k-mer errors were detected, representing an exceptionally high assembly accuracy.

‡ Breadth of coverage and MAPQ were calculated using minimap2 (ver. 2.31) and SAMtools (ver. 1.23.1). MAPQ score of 60 indicates that the reads were uniquely and reliably mapped across the genome at the highest confidence level.

§ Genome coverage was calculated by dividing the total bases of raw reads by the genome size (the number of genomic bases).

Comparison of 16S rRNA gene sequences revealed that the strain NHSK-567 showed 96.52% sequence similarity to *Hydrogenobacter thermophilus* TK-6^T^ (AP011112.1), 95.74% to *Pampinifervens diazotrophicum* T-2^T^ (CP117180.1) and 94.37% to *Thermocrinis ruber* OC 1/4^T^ (CP007028.1). The calculated dDDH and orthoANI values are summarized in Table 2.

**Table 2.** Genomic relatedness of strain NHSK-567 against closely related type strains

Strain	16S rRNA gene sequence similarity (%)^*^	dDDH value (%)^†^	ANI value (%)^‡^
*H. thermophilus* TK-6^T^	96.38	18.6 (16.4–21.0)	71.41
*P. diazotrophicum* T-2^T^	95.80	17.8 (15.7–20.1)	73.34
*T. ruber* OC 1/4^T^	94.96	18.1 (15.9–20.4)	71.17

* 16S rRNA gene sequence similarity (%) was calculated using the Pairwise Nucleotide Sequence Alignment for Taxonomy tool in EzBioCloud.

† dDDH values (%) were calculated using formula 2 of the GGDC on the TYGS. Values (%) in parentheses represent the 95% CIs.

‡ orthoANI values (%) were calculated on the EzBioCloud web server.

The genomic data from the novel strain will enable a more detailed analysis of its physiological traits and provide new insights into the ecology and evolution of thermophiles and chemolithotrophs.

## Supplementary material

10.1099/acmi.0.001222.v4 Supplementary Material 1.

10.1099/acmi.0.001222.v4 Supplementary Material 2.

## Abbreviations

dDDH: digital DNA–DNA hybridization
GGDC: Genome-to-Genome Distance Calculator
IMR: Integrated Microbiome Resource
MAPQ: mean mapping quality
orthoANI: orthologous average nucleotide identity
QV: quality value
TYGS: Type (Strain) Genome Server
