# Supplementary material for: Complete genome sequence of Aquificaceae bacterial strain NHSK-567, isolated from Nakabusa Hot Springs, Japan
Source: Access Microbiol. 2026 Nov 5;8(11):001222.v4. doi: 10.1099/acmi.0.001222.v4 (PMC13752024; doi:10.1099/acmi.0.001222.v4)

Supplementary Material 2: Galaxy workflow for genome analysis for NHSK-567

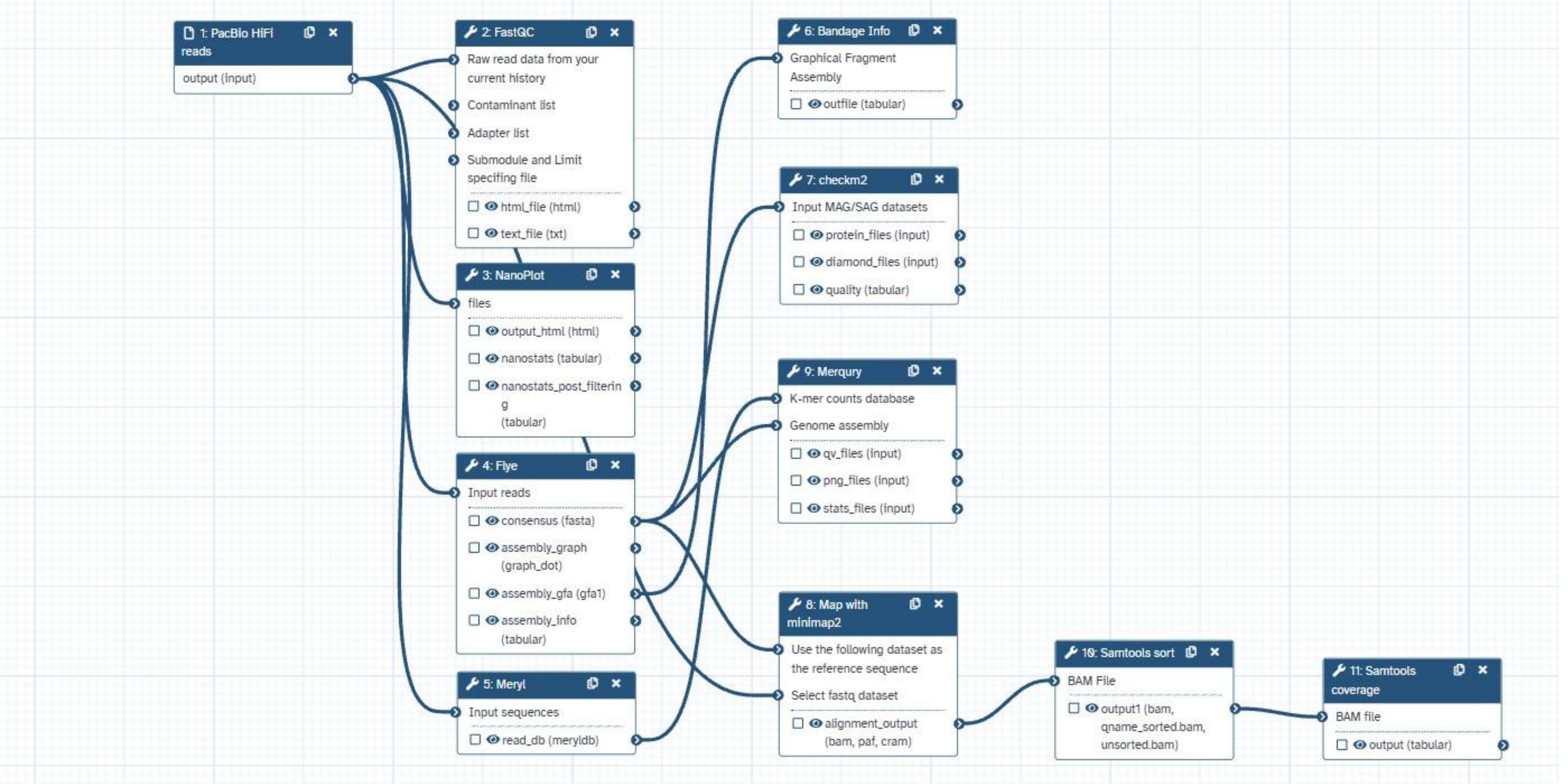

Supplement: Supplementary file 1 [file acmi-8-01222-s002.pdf]
